# Fatal and Rapid Progressive Isolated Cerebral Mucormycosis Involving the Bilateral Basal Ganglia: A Case Report

**DOI:** 10.3389/fneur.2020.00295

**Published:** 2020-04-21

**Authors:** Gao-jia Zhang, Shao-ke Zhang, Zan Wang, Yi-xin Zhu, Jun Kong, Li-li Huang, Yi-jing Guo, Yan-juan Wang, Rong-cheng Zou, Chun-ming Xie

**Affiliations:** ^1^Department of Neurology, Nanjing Lishui People's Hospital, Nanjing, China; ^2^School of Medicine, Southeast University, Nanjing, China; ^3^Department of Neurology, Affiliated Zhongda Hospital, Southeast University, Nanjing, China; ^4^Department of Neurosurgery, Affiliated Zhongda Hospital, Southeast University, Nanjing, China; ^5^Department of Intensive Care Unit, Affiliated Zhongda Hospital, Southeast University, Nanjing, China

**Keywords:** isolated cerebral mucormycosis, *Rhizopus microspores*, histopathological examination, cerebrospinal fluid, next-generation sequencing technology

## Abstract

Isolated cerebral mucormycosis is a clinical type of mucormycosis that is estimated to account for 8% of all mucormycosis cases. The clinical symptoms of isolated cerebral mucormycosis are elusive, and thus conventional techniques often lake sensitivity and specificity. Moreover, cultures are often negative, even when direct microscopy examination is positive. Although histopathology will probably remain the gold standard for the diagnosis of mucormycosis, obtaining a biopsy specimen is not always feasible in most vulnerable populations. Thus, molecular approaches are currently used as an advantageous assistant examination method to improve the early identification of the causative agent and subsequently guide therapy to improve the prognosis of patients. Here, we report a case of isolated cerebral mucormycosis caused by *Rhizopus microspores* in a healthy young adult that was identified using next-generation sequencing technology.

## Introduction

The global burden of fungal diseases is increasing due to the growing number of people at risk for fungal infections and the changes in the geographical distribution of fungi (1). Mucormycosis often presents as a rare, fatal opportunistic fungal infection that is caused by members of the Mucorales family including the most frequent species *Rhizopus*, followed by *Mucor Rhizomucor, Absidia, Apophysomyces, Cunninghamella*, and *Saksenaea* (2, 3). Mucorales are able to downregulate several host defense mechanisms. Researchers have identified the specific receptors through which Mucorales attach to the endothelium, facilitating their endocytosis and subsequent angioinvasion (4). A defining characteristic of the pathophysiology of this infection is tissue infarction, angioinvasion with thrombosis, and tissue necrosis (3). Isolated cerebral mucormycosis (ICM) is a clinical types of mucormycosis that is estimated to account for 8% of all mucormycosis cases (5). The clinical symptoms of ICM are elusive, and conventional techniques often lake sensitivity and specificity; in particular, cultures are often negative despite positive direct microscopy examination (6–10). Indeed, the positive culture rate is only 50% for all types of mucormycosis with a confirmed pathological diagnoses (5); the positive culture rate is lower for cerebral mucormycosis, at only 38% (11). Although histopathology will probably remain the gold standard for the diagnosis of mucormycosis (12), obtaining a biopsy specimen is not always feasible in most vulnerable populations. Thus, molecular approaches are currently used as an adjuvant examination method to improve the early identification of the causative agent and subsequently guide therapy to improve the prognosis of patients. Here, we report a case of ICM caused by *Rhizopus microspores* in a healthy adult, which was identified using Next-generation sequencing (NGS) technology.

## Methods

NGS technology-based pathogen detection of the patient's cerebrospinal fluid (CSF) sample was approved by his parents and primary care physician. CSF samples were processed in a medical laboratory according to the NGS assay manual (BGISEQ-50). The general detection process was as follows: (1) 600 μl CSF was used for extraction of nucleic acids to prepare cDNA libraries, and (2) sequencing was performed with a BGISEQ50 after the library was validated with a 2100 Bioanalyzer system (Agilent Technologies, Inc.) and Qubit (ABI Life Technologies). The bioinformatics process mainly included the following steps: (1) host reads were subtracted; and (2) the remaining reads were aligned to a reference database composed of multiple public sequence resources of bacteria, viruses, fungi and parasites.

## Case Report

**Figure 1:**
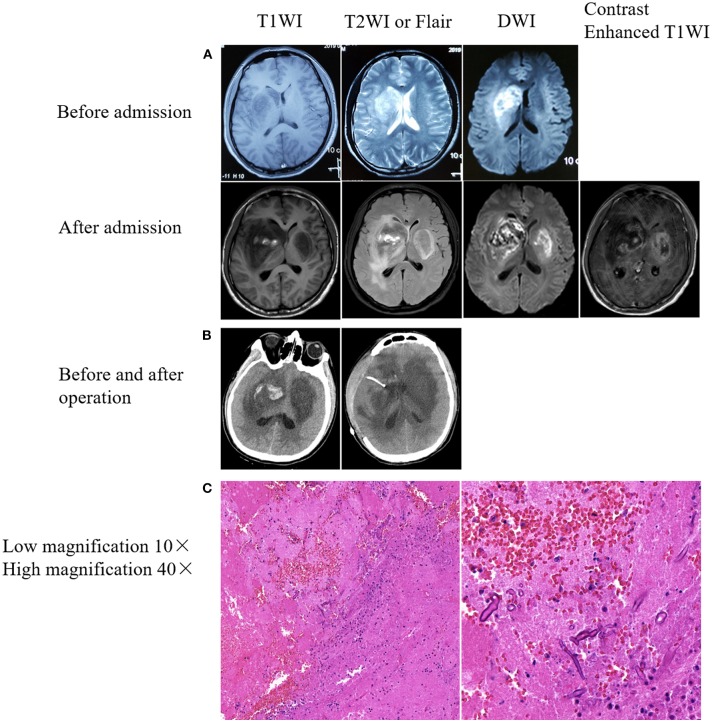
Brain imaging and immunohistochemical results. **(A)** Rapid progressive damage in the bilateral basal ganglia. Upper: Right basal ganglia lesion with patchy abnormal signals before admission. Lower: The bilateral basal ganglia showed patchy abnormal signals after admission. Bilateral basal ganglia lesions with hemorrhage on the right side. **(B)** Mixed density shadow in the bilateral basal ganglia with peripheral parenchymal edema and postoperative resection of right basal ganglia lesions. **(C)** Pathological results from the brain tissues of lesions in the right basal ganglia. Hematoxylin-eosin staining. Fungal spores and hyphae were observed in a small amount of necrotic brain tissue.

Male, 27 years old, woodworker, who was admitted to the Department of Neurology of Zhongda Hospital on April 18th, 2019, because of a cough with fever for 12 days and headache for 4 days. Twelve days prior to admission, he presented with a cough and fever of up to 38.0°C, without expectoration or headache, and accepted antibiotic treatment for 3 days at a community-based hospital (specific medication was unknown). The cough and fever symptoms did not improve. Then he returned to his hometown. The doctors at the local community hospital administered azithromycin intravenously. At the same time, oral antipyretics were administered. Four days prior to hospital admission, the patient developed a headache and his temperature increased to 39.0°C, accompanied by nausea and vomiting. One day later, he was admitted to Xuzhou First People's Hospital. Brain magnetic resonance imaging (MRI) showed space-occupying lesions in the right basal ganglia, with obvious local edema (Figure 1A, before admission). Blood routine: white blood cells (WBC) 15.27 ^*^ 10^9^/L, neutrophils 67.8%. After treatment with an antibiotic, the symptoms worsened, and his left limb muscle strength decreased. For further treatment, he was transferred to the NICU of the Department of Neurology of Zhongda Hospital on April 18. The patient was previously healthy, had a history of smoking for 10 years (10 cigarettes/day), had no history of immunosuppressive drug use, and had no other special abnormalities. On examination, Temperature 37.8°C; heart rate 66 beats/ min; respiratory rate 18 breaths/min; blood pressure, 107/62 mmHg; weight, 60 kg; and body mass index, 20.8 kg/m^2^. General examination showed no lesions of the nose, paranasal sinuses, orbits, or skin. Physical examination showed normal consciousness, left-sided central facial paralysis, muscle strength four out of five in the left upper and lower limbs, and the left-sided pathological reflex (Babinski sign) and meningeal irritation were positive. Blood routine tests were reexamined: WBC 15.42 ^*^ 10^9^/L; neutrophil 12.92 ^*^ 10^9^/L; neutrophil ratio 83.81%; lymphocyte ratio 9.32%. Lumbar puncture was operated: CSF pressure 220 mm H_2_O, light yellow, total cell count: 160 ^*^ 10^6^/L; white cell count: 60 ^*^ 10^6^/L; red blood cell count: 100 ^*^ 10^6^/L, lobulated nucleus cells: 40%; monocyte: 60%. Biochemistry of CSF: Cl^−^: 123.8 mmol/L (120.0 ~ 130.0 mmol/L), glucose: 1.96 mmol/L (2.50 ~ 4.50 mmol/L), protein: 2557.9 mg/L (120 ~ 600 mg/L). No bacteria or fungi were found in blood, sputum and urine cultures. Tests for viruses were performed, with no positive results (including hepatitis B virus, hepatitis C virus, human immunodeficiency virus, treponema pallidum). The patient was empirically treated with meropenem (2 g, q8h) because of concern for bacterial meningoencephalitis. Glycerol fructose and mannitol were also used to reduce intracranial pressure. On the night of admission, a brain plain scan and enhanced MRI showed lesions in the right basal ganglia and frontotemporal lobe, and a new lesion in the left basal ganglia was found (Figure 1A, after admission). Magnetic resonance angiography of the head did not reveal evidence of stenosis or thrombosis of any major intracranial blood vessels. We then called the consultation of a neurosurgeon, and a diagnosis of brain abscess was made. Considering the incomplete formation of the abscess capsule, conservative treatment was recommended. We then adjusted our treatment plan as follows: imipenem (0.5 g, q6h), vancomycin (1,000 mg, q12h), and ornidazole (0.5 g, q12h). On the second day of admission, the patient developed altered consciousness, Glasgow Coma Score (GCS) of 5 (1 + 1 + 3). The diameter of the bilateral pupils was not equal: 3.5 mm on the right side, 2.1 mm on the left side, indicative of symptoms of cerebral hernia. Brain and chest computed tomography (CT) examinations revealed bilateral basal ganglia lesions with a right basal ganglia hemorrhage broken into the ventricle (Figure 1B, left) no abnormalities were found by chest CT. We then contacted the neurosurgeon again. Under general anesthesia, the patient underwent an emergency decompressive craniectomy and resection of the lesion of the right basal ganglia. A large amount of gray-yellow-red brain tissues with dark red blood clots was observed, and some brain tissues were removed for pathological biopsy. After surgery, the patient was transferred to ICU. Meropenem combined with vancomycin for anti-infective treatment was continued, and mannitol was also continued to reduce intracranial pressure. Endotracheal intubation and ventilator-assisted breathing therapy were also administered. On the same day, the CSF in the ventricular drainage tube was examined. No bacteria or acid-fast bacilli were detected by Gram staining. On the third day, the patient was still comatose, with a GCS score of 5 (1 + 1 + 3). The body temperature remained high, reaching 38.8°C. On the fourth day, we stopped meropenem and ceftriaxone was administered empirically. Meanwhile, we carried out NGS of the CSF etiology. The symptoms of cerebral hernia worsened further: bilateral pupil diameter increased, right pupil diameter 5 mm, left pupil diameter 3 mm. The light reflex disappeared. Re-examination of the brain CT showed that brain tissue swelling was more severe than before (Figure 1B, right). On the sixth day, the brain tissue histopathological results showed a large number of infiltrated neutrophils and abscesses formation, and fungal hyphae and spores were found in surrounding necrotic brain tissues, which tended to be Mucor (Figure 1C). Meanwhile, the results of NGS were obtained, suggesting Rhizopus microspore infections. No bacteria, viruses, mycoplasma, chlamydia, *Mycobacterium tuberculosis* complex or other pathogenic microorganisms were detected. We then stopped ceftriaxone and vancomycin, and added amphotericin B lipid formulation (5 mg/kg) and posaconazole (200 mg q6h). Unfortunately, the patient's condition continued to deteriorate during the night of April 24th. The bilateral pupil diameters both increased to 5 mm, and the light reflex completely disappeared. On the seventh day, the patient's family members stopped treatment, and the patient died on the sixth day after discharge.

## Discussion

Mucormycosis is a disease caused by Mucorales infection and has gained attention over the past few decades because of its increasing incidence in immunosuppressed populations (4). Mucorales, fast-growing thermotolerant fungi that are widely distributed in the environment, are commonly found in decaying organic material or agricultural and forest soils (13). Although exposure to these organisms occurs every day, their low virulence causes them to seldom lead to disease (14).

ICM is seldom reported (9, 15–19), especially in healthy individuals (15, 20, 21). The special characteristics of the case reported herein are mainly manifested in the following aspects. Mucormycosis often occurs in patients with immunosuppression, such as patients with hematologic malignancies, neutropenia, or diabetic ketoacidosis, those who have undergone organ transplantation, and intravenous drug abusers (9, 22). However, the patient did not have any relevant medical history. Extensive examinations did not reveal a systemic disease or predisposing condition after admission. Previous reports have shown that patients with ICM often have a history of intravenous drug abuse (6, 10, 11, 15). Possibly because the fungal spores are inoculated directly into the blood through contaminated illicit drugs. Some microspores might escape pulmonary capillary filtration, and then enter the arterial circulation and eventually the intracranial structure (9). The patient's family members indicated that the patient had no relevant medical history. No syringe needle eyes or ulcerations were observed on the patient's skin. Extensive examinations did not reveal any systemic diseases such as primary immunodeficiency, acquired immunodeficiency syndrome, tuberculosis, or tumors. We think the cause may be as follows. First, the patient was a carpenter, working in a poor environment and surrounded by decaying wood debris and therefore had more opportunities to make contact with fungi. At the same time, the patient had worked overtime and stayed up late for a couple of days prior to symptom onset, which may have suppressed his immune system and made him more susceptible to the pathogen. Finally, the patient's first symptoms were accompanied by a cough, which was manifested from a respiratory tract infection. It is speculated that the fungus probably penetrated the brain via some other unapparent localization (likely the nasopharyngeal mucosa or upper air passages), where it was transferred to the brain via the blood or lymph.

Second, ICM has been shown to have consistent predilection for the basal ganglia, and Mucorales usually invade the basal ganglia on one side, and then rapidly invade the contralateral basal ganglia in a short time (6, 11), as reported in our case. Kerezoudis's recent summary of ICM found that more than 71% of cases involved the basal ganglia (11). The basal ganglia lesions are probably caused by hematogenous seeding through perforating branches of the middle cerebral artery (11). This tendency to destroy the basal ganglia can be explained by the angioinvasive nature of the organism. The hyphae proliferate in the internal elastic lamina, penetrate the endothelium, and eventually occlude the vascular lumen (9). In our case, after unilateral basal ganglia involvement, the fungus rapidly extended to the contralateral basal ganglia. Brain MRI showed patchy abnormal signal shadows in the bilateral basal ganglia, inhomogeneous diffusion limitation, low signal on T1WI, a mixed, slightly high signal on T2WI and multiple patchy high signals on the right basal ganglia. Heterogeneous enhancement was found at the edge of the lesion. Although cerebral mucomycosis has no unique imaging characteristics (11), the possibility of ICM should be considered for rapidly enlarged brain abscesses in the basal ganglia, even if the patient has no risk factors associated with mucormycosis.

Finally, the mortality and disability rates of ICM are extremely high, and up to 65% of patients die (11). The survival rate can be improved by making a definite diagnosis as soon as possible and using amphotericin B and surgical debridement to remove infected tissues as early as possible (10, 11, 15, 18). The gold standard for the diagnosis of mucormycosis is to detect invasive mycelia on tissue biopsy (12). However, obtaining a biopsy specimen is not always feasible (12) and blood and CSF cultures are often negative (6, 7). Therefore, finding a non-invasive, rapid and reliable diagnostic test is a major obstacle in the management of ICM. Molecular methods are effective for identifying Mucorales and may be able to diagnose pathogenic microorganisms at the species level (13), which is often impossible in tissue biopsy (11). Because of the life-threatening cerebral hernia during the course of treatment, we had to perform a decompressive craniectomy and resection of the lesion of the right basal ganglia. A biopsy was performed on the excised pathological brain tissue. Subsequently, we found a small number of fungal spores and mycelia on the brain tissue lesion, most likely of the Mucorales order. However, using NGS technology, *Rhizopus microsporus, Mucor racemosus, Cladosporium subnodosum*, and *Mucor indicus* were found, with the gene sequence of *Rhizopus microsporus* being the most prominent, followed by *Mucor*. No bacteria, viruses, mycoplasma, chlamydia, parasites or *M. tuberculosis* were found, suggesting that this method has high sensitivity and specificity. Therefore, for patients suspected of intracranial infection, pathogenic NGS technology in CSF performed at an early stage may help identify the pathogen as quickly as possible.

Amphotericin-B is the most active antifungal agent against mucormycosis, and the timely initiation of amphotericin-B lipid formulation monotherapy constitutes its first-line treatment (23). The standard dose of amphotericin-B lipid formulation is 5 mg/kg (23). After definite etiological diagnosis, we immediately used AMB lipid formulation (5 mg/kg) and Posaconazole (200 mg q6h) 18 days after symptoms began to appear and 9 days following signs of intracranial infection. Unfortunately, after 2 days of continuous antifungal treatment, the patient's symptoms did not improve, and he was discharged automatically. He was subsequently sent to a local hospital to continue antifungal treatment and died on the sixth day after discharge.

In the process of diagnosing and treating this patient, a lumbar puncture was performed on the first day of admission. If we had carried out NGS immediately, we could have identified the specific pathogenic microorganisms on the third day of the genetic examination, that is, 15 days after the onset of the disease and 7 days after the emergence of the intracranial infection symptoms. Thus, targeted antifungal drugs would have been administered 3 ~ 4 days earlier, and the patient's outcome may have been different.

## Conclusion

In summary, for patients suspected of intracranial infection, especially considering Mucorales infection, NGS and other molecular techniques may be effective and should be considered as adjuvants to traditional diagnostics to achieve early diagnosis and treatment, particularly when the gold standard is inconclusive or not feasible.

## Data Availability Statement

The datasets generated for this study are available on request to the corresponding author.

## Ethics Statement

Written informed consent was obtained from the individual(s) for the publication of any potentially identifiable images or data included in this article.

## Author Contributions

During the hospitalization of this patient at Zhongda Hospital, RZ was refresher doctor of NICU, YW was a resident in the NICU, YG was the chief physician for the NICU, LH was the attending doctor in the Department of Intensive Care Unit, JK of the Department of Neurosurgery performed the emergency surgery, YZ was the attending doctor in the NICU. All of these physicians provided resources for this patient. GZ collected the clinical data and wrote the first draft of the manuscript. SZ collected the clinical data. ZW revised the manuscript. CX revised the manuscript and provided the funding. All authors commented on previous versions of the manuscript. All authors read and approved the final manuscript.

## Conflict of Interest

The authors declare that the research was conducted in the absence of any commercial or financial relationships that could be construed as a potential conflict of interest.
